# Troponin Elevation on Admission Along With Dynamic Changes and Their Association With Hemorrhagic Transformation After Thrombolysis

**DOI:** 10.3389/fnagi.2021.758678

**Published:** 2021-10-13

**Authors:** Zicheng Cheng, Zhenxiang Zhan, Xiaoyan Huang, Lingfan Xia, Tong Xu, Zhao Han

**Affiliations:** Department of Neurology, The Second Affiliated Hospital and Yuying Children’s Hospital of Wenzhou Medical University, Wenzhou, China

**Keywords:** troponin, hemorrhagic transformation, acute ischemic stroke, thrombolysis, alteplase

## Abstract

**Background:** Hemorrhagic transformation (HT) is a common complication of intravenous thrombolysis with alteplase. Cardiac troponin has been found to be associated with poor prognosis and cognitive impairment in acute ischemic stroke. But studies on the relationship between troponin and HT after thrombolysis are scarce.

**Methods:** This study retrospectively analyzed thrombolytic patients from June 2015 to June 2021 in the Second Affiliated Hospital of Wenzhou Medical University. Cardiac troponin I were measured on admission and on following days to determine the presence of elevation and dynamic changes. HT within 24–36 h after treatment was identified by cranial computed tomography (CT). Besides, a score on the modified Rankin Scale (mRS) > 2 at discharge was defined as unfavorable outcome. Univariate analysis was used to explore the factors related to the troponin elevation on admission and troponin dynamic changes. Multivariate logistic regression model was used to investigated the association between troponin elevation on admission, troponin dynamic changes and HT after thrombolysis, respectively.

**Results:** Troponin levels on admission were measured in 377 patients, and follow-up assay was performed in 292 patients (77.5%). 39 patients (10.3%) had troponin elevation on admission, and 66 patients (22.6%) had troponin dynamic changes comprising rising and falling pattern. The pre-existing heart disease, renal insufficiency and higher stroke severity are related to both troponin elevation on admission and the subsequent troponin dynamic changes. After adjusting the potential confounding factors, logistic regression model showed that patients with troponin elevation on admission had insignificant trend to develop HT (OR 2.23, 95%CI 0.96–5.21, *p* = 0.063), while patients with troponin dynamic changes had significantly higher risk of HT (OR 2.27, 95%CI 1.06–4.85, *p* = 0.034). Compared to the troponin elevation, a statistically stronger association was present between rising troponin dynamic changes and unfavorable outcome (OR 2.20, 95%CI 1.05–4.60, *p* = 0.037).

**Conclusion:** Troponin dynamic changes are associated with HT after thrombolysis. Serial measurements are quite necessary in thrombolytic patients with risk factors associated with troponin dynamic changes (e.g., advanced age, pre-existing heart disease, higher NIHSS score, and troponin elevation on admission).

## Introduction

Intravenous thrombolysis with alteplase effectively improves functional outcome in patients with acute ischemic stroke (AIS) (Emberson et al., 2014) and, currently, is the most widely prescribed ultra-early treatment for AIS in all grades of hospitals. Nonetheless, thrombolytic therapy also brings some adverse effects, especially hemorrhagic transformation (HT). As a common complication of thrombolysis, HT can causes early deterioration of neurological function, early death and poor long-term prognosis (Park et al., 2012; Yaghi et al., 2017). Although a number of blood biomarkers have been found to be relevant indicators of HT, further exploration is still needed to perfect them (Lu et al., 2018).

Cardiac troponins, a specific marker for myocardial injury, are recommended by the American Heart Association (AHA)/American Stroke Association (ASA) for routine detection in all patients with AIS (Powers et al., 2019). Troponin elevation is frequently detected in AIS due to premorbid cardiac disease, comorbid acute coronary syndrome (ACS) and stroke–heart syndrome (Scheitz et al., 2021). There is strong evidence that troponin elevation is associated with poor short- and long-term outcome (Scheitz et al., 2012, 2014; Ahn et al., 2017; Wrigley et al., 2017) and cognitive impairment (Broersen et al., 2020). However, few studies have reported the relationship between troponin and HT (Liu et al., 2016a), particularly secondary to thrombolysis.

Therefore, the aim of our study was to investigate troponin and its association with HT after thrombolysis in patients with AIS. In view of the dynamic changes of troponin levels in the course of AIS (Anders et al., 2013; Scheitz et al., 2014), we adopted two indexes, that is troponin elevation on admission and troponin dynamic changes.

## Materials and Methods

This study was a single-center observational study in the Second Affiliated Hospital of Wenzhou Medical University. We retrospectively analyzed the consecutive patients with AIS who received intravenous thrombolysis in our hospital from June 2015 to June 2021. Patients treated with 0.9mg/kg (maximum 90mg) alteplase within 4.5 h of onset were included. Patients with the following conditions were excluded: (1) troponin levels were not measured on admission, (2) cranial computed tomography (CT) was not repeated within 24–36 h, (3) comorbid ACS, identified mainly by symptoms and electrocardiographic findings, and (4) thrombolysis with low-dose alteplase or interruption of thrombolysis for reasons other than HT. This study was approved by the Ethics Committee of the Second Affiliated Hospital and Yuying Children’s Hospital of the Wenzhou Medical University. Written informed consent was obtained from participants or their guardians.

### Data Collection

We collected data regarding the patient’s demographics (i.e., age and sex), vascular risk factors, comorbidities, clinical examination findings, blood test results, cranial CT, echocardiography, electrocardiogram and therapeutic measures. Vascular risk factors comprised hypertension, diabetes, hyperlipidemia, and smoking, while comorbidities comprised atrial fibrillation (AF), coronary artery disease (CAD), previous stroke. Clinical examination findings consisted of blood pressure on admission and stroke severity assessed using the National Institutes of Health Stroke Scale (NIHSS) score. Blood test results included troponin, glucose, HbA1c, platelet, international normalized ratio (INR), creatinine, total cholesterol (TC), low-density lipoprotein cholesterol (LDL-C). Treatment measures involved ongoing antithrombotic therapy, onset-to-treatment time (OTT), and bridging therapy. Stroke etiology was evaluated according to the Trial of Org 10172 in Acute Stroke Treatment (TOAST) classification (Adams et al., 1993).

Cardiac troponin I levels were measured with AccuTnI + 3 assay on chemiluminescnet analyzer Access2 (Beckman Coulter, United States). The first blood sample was taken preceding treatment in the emergency department, and the majority of patients underwent retesting on the following days. For this assay, the lower limit of detection was 12 ng/L and the 99th percentile of the upper reference limit (URL) was 34 ng/L. Values above the URL were considered troponin elevation. Troponin dynamic changes were defined as a rise or fall of more than 20% in troponin levels and at least one troponin I value above the URL on serial measurements (Thygesen et al., 2018).

### Outcome Assessment

All patients received routine cranial CT examination at admission and reviewed within 24-36 h after treatment. HT was defined as bleeding observed on follow-up CT, which was not observed on first CT examination. According to the European Cooperative Acute Stroke Study (ECASS) criteria, we classified HT into hemorrhagic infarction (HI-1 or-2) and parenchymal hemorrhage (PH-1 or-2) (Hacke et al., 1995). All CT images were reviewed by two independent neurologists and discussed when disagreement was encountered. The inter-rater agreement was estimated using Kappa test. Besides, functional neurological outcome was assessed using the modified Rankin Scale (mRS) at discharge. A score on the mRS > 2 was defined as unfavorable outcome.

### Statistical Analysis

Normally distributed continuous variables were presented as mean ± SD, skewed continuous variables as median (interquartile range), and categorical variables as absolute values (percentages). For univariate analysis, differences between continuous variables in two groups were performed by *t*-test or *U*-test as appropriate, while differences between categorical variables in two or more groups were performed by Pearson’s chi-square test or Fisher’s exact probabilities test as appropriate.

Univariate and multiple logistic regression models were used to analyze the association between troponin elevation on admission, troponin dynamic changes, and HT after thrombolysis, respectively. For the multiple model, model 1 was adjusted for age and sex, and model 2 was adjusted for age, sex and other variables with *p* < 0.1 in the univariate analysis. Subgroup analysis was executed to further investigate the relationship between troponin and HT after thrombolysis in different types of patients. All statistical tests were two-tailed, and *p* < 0.05 was considered statistically significant. All analyses were performed using SPSS 25.0 (IBM, Armonk, NY, United States) and figures were drawn with the use of Excel software 2019 (Microsoft, Redmond, WA, United States).

## Results

A total of 408 patients with acute ischemic stroke were treated with thrombolysis from June 2015 to June 2021 in our hospital. Based on the protocol (Supplementary Figure 1), 31 patients were excluded, and finally 377 patients were included in the analysis. The median age was 71 (61-81) years, and 250 patients (66.3%) were male patients. Troponin elevation on admission was present in 39 patients (10.3%). Serial measurements were performed in 292 patients (77.5%), and among these, 66 patients (22.6%) had dynamic changes, with a rise pattern in 56 and a fall pattern in 10 patients. No significant difference was found between patients with rising and falling pattern. The median interval between serial measurements was 24h (12h–28h). Patients who had serial measurements had a higher proportion of CAD, higher NIHSS scores, and lower platelet count (all *p* < 0.05, Supplementary Table 1).

Patients with troponin elevation on admission had more comorbid AF and CAD, cardiac dysfunction (lower ejection fraction), renal insufficiency (higher serum creatinine) and higher stroke severity (higher NIHSS scores) than those without troponin elevation (all *p* < 0.05, Supplementary Table 2). In addition to the above factors, patients who developed troponin dynamic changes were prone to advanced age, female and having higher INR compared to those without dynamic changes (all *p* < 0.05, Table 1). Patients with troponin elevation on admission were also more likely to have subsequent dynamic changes (*p* < 0.001).

**TABLE 1:** Clinical characteristics of patients, stratified by the presence of troponin dynamic changes.

	**Troponin dynamic changes**		***P***
	**Yes (*n* = 66)**	**No (*n* = 226)**	
Age, years (IQR)	81 (69–83)	69 (61–79)	** < 0.001**
Male, n (%)	34 (51.5%)	158 (69.9%)	**0.006**
**Vascular risk factors**			
Hypertension, n (%)	52 (78.8%)	173 (76.5%)	0.70
Diabetes mellitus, n (%)	15 (22.7%)	78 (34.5%)	0.071
Hyperlipidemia, n (%)	24 (36.4%)	85 (37.6%)	0.85
Current Smoking, n (%)	15 (22.7%)	53 (23.5%)	0.90
**Comorbidities**			
Atrial fibrillation, n (%)	36 (54.5%)	67 (29.6%)	** < 0.001**
Coronary artery disease, n (%)	12 (18.2%)	17 (7.5%)	**0.011**
Previous stroke, n (%)	12 (18.2%)	24 (10.6%)	0.10
**Treatment status**			
OTT, min (mean ± SD)	157.8 ± 54.4	167.4 ± 56.7	0.24
Ongoing antithrombotic therapy, n (%)	12 (18.2%)	32 (14.2%)	0.42
Bridge therapy, n (%)	12 (18.2%)	30 (13.3%)	0.32
NIHSS score (IQR)	14 (9–18)	6 (4–12)	** < 0.001**
Baseline SBP, mmHg (mean ± SD)	161.8 ± 25.3	159.4 ± 22.5	0.45
Baseline DBP, mmHg (mean ± SD)	88.3 ± 18.9	88.9 ± 15.8	0.79
Ejection fraction,% (IQR)	62 (58–67)	65 (61–68)	**0.001**
HT, n (%)	21 (31.8%)	30 (13.3%)	** < 0.001**
**Laboratory test**			
Troponin elevation on admission, n (%)	26 (39.4%)	7 (3.1%)	** < 0.001**
Baseline blood glucose, mmol/L (IQR)	6.88 (5.88–9.16)	7.19 (6.09–9.35)	0.35
Platelet, 10^9^/L (IQR)	191 (158–226)	189 (165–227)	1.00
INR (IQR)	1.06 (1.00–1.12)	1.03 (0.98–1.09)	**0.011**
Creatinine, umol/L (IQR)	84 (65–105)	69 (59–83)	** < 0.001**
HbA1c,% (IQR)	5.70 (5.45–6.10)	5.91 (5.50–6.68)	0.065
TC, mmol/L (IQR)	4.11 (3.61–4.96)	4.22 (3.69–5.12)	0.43
LDL_C, mmol/L (IQR)	2.33 (1.87–3.17)	2.58 (2.11–3.34)	0.11
**Stroke etiology**			**0.002**
Large artery atherosclerosis	18 (27.3)	69 (30.5)	
Cardioembolism	36 (54.5)	67 (29.6)	
Small vessel occlusion	5 (7.6)	54 (23.9)	
Other determined	1 (1.5)	8 (3.5)	
Undetermined	6 (9.1)	28 (12.4)	

*DBP, diastolic blood pressure; HT, hemorrhagic transformation; INR, international normalized ratio; IQR, interquartile range; LDL-C, low-density lipoprotein cholesterol; NIHSS, National Institutes of Health Stroke Scale; OTT, onset-to-treatment time; SBP, systolic blood pressure; SD, standard deviation; TC, total cholesterol. The bold values represent significant results in univariate analysis (*P* < 0.05).*

**FIGURE 1:**
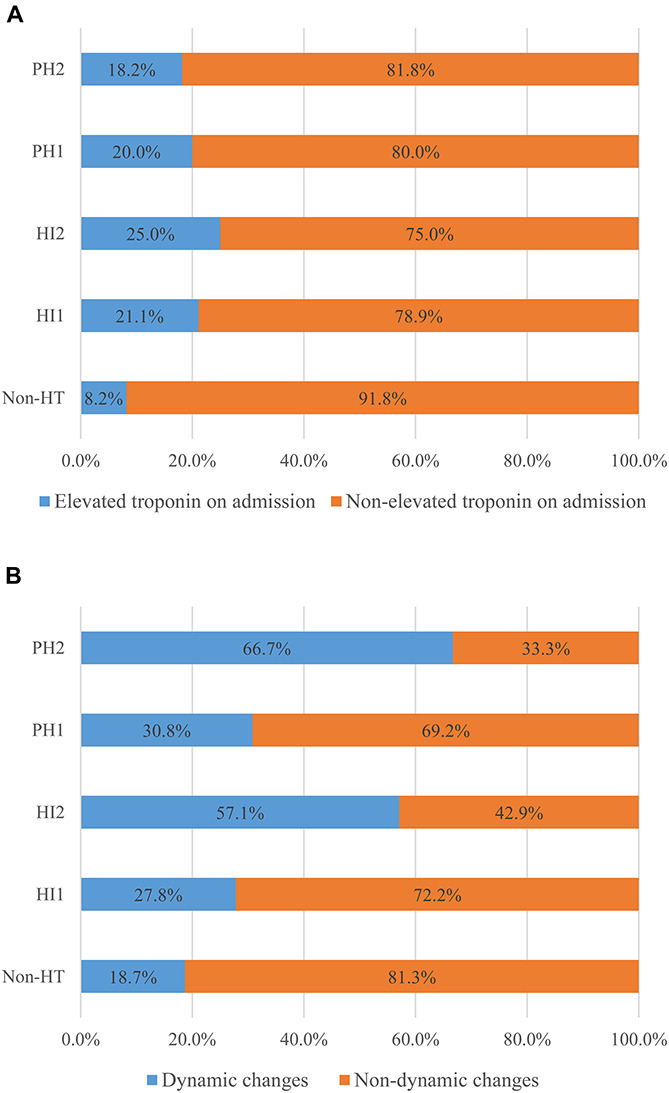
Differences in troponin elevation on admission **(A)** and troponin dynamic changes **(B)** between groups with non-HT and other HT types.

**FIGURE 2:**
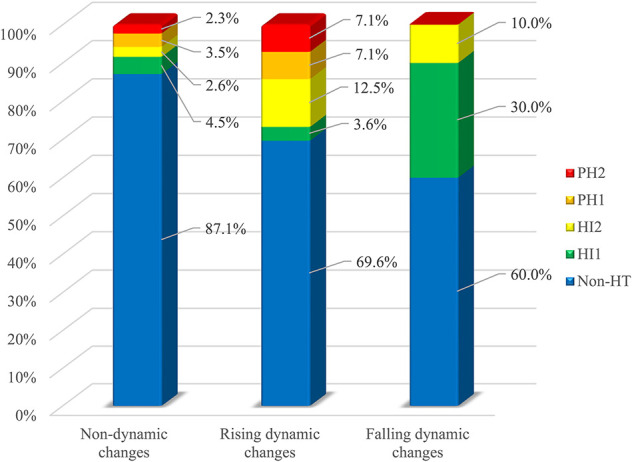
Differences in HT between groups with non-dynamic, rising and falling dynamic changes.

Excellent inter-rater agreement for HT (κ = 0.94) and its further classification (κ = 0.84) was observed. Eventually, HT was identified in 61 patients (16.2%), comprising 19 (5%) with HI-1, 16 (4.2%) with HI-2, 15 (4.0%) with PH-1, and 11 (2.9%) with PH-2. Univariate analysis showed that patients with HT had a higher proportion of troponin elevation on admission, troponin dynamic changes and AF as well as higher NIHSS score, INR, and lower ejection fraction (all *p* < 0.05, Table 2). As shown in Figure 1, compared with patients without HT, any HT type had higher proportion of troponin elevation on admission and troponin dynamic changes. The proportion of troponin elevation on admission was the highest in HI -2, while the proportion of troponin dynamic changes was the highest in PH-2. Figure 2 shows that patients with either rising or falling changes had a higher risk of HT compared to patients without dynamic changes (*p* = 0.001), but patients with falling changes developed only HI rather than PH.

**TABLE 2:** Clinical characteristics of patients, stratified by the presence of HT.

	**HT (*n* = 61)**	**Non-HT (*n* = 316)**	***P***
Age, years (IQR)	73 (61–82)	70 (61–80)	0.37
Male, n (%)	39 (63.9%)	211 (66.8%)	0.67
**Vascular risk factors**			
Hypertension, n (%)	48 (78.7%)	241 (76.3%)	0.68
Diabetes mellitus, n (%)	17 (27.9%)	98 (31.0%)	0.63
Hyperlipidemia, n (%)	19 (31.1%)	118 (37.3%)	0.36
Current Smoking, n (%)	17 (27.9%)	73 (23.1%)	0.42
**Comorbidities**			
Atrial fibrillation, n (%)	38 (62.3%)	89 (28.2%)	** < 0.001**
Coronary artery disease, n (%)	8 (13.1%)	22 (7.0%)	0.17
Previous stroke, n (%)	8 (13.1%)	37 (11.7%)	0.76
**Treatment status**			
OTT, min (mean ± SD)	159.8 ± 56.1	165.9 ± 57.2	0.45
Ongoing antithrombotic therapy, n (%)	12 (19.7%)	41 (13.0%)	0.17
Bridge therapy, n (%)	11 (18.0%)	37 (11.7%)	0.18
NIHSS score (IQR)	13 (7–18)	7 (4–13)	** < 0.001**
Baseline SBP, mmHg (mean ± SD)	159.3 ± 27.1	160.7 ± 23.4	0.68
Baseline DBP, mmHg (mean ± SD)	91.7 ± 17.8	88.9 ± 16.4	0.23
Ejection fraction,% (IQR)	63 (59–67)	64 (61–68)	**0.017**
**Laboratory test**			
Troponin elevation on admission, n (%)	13 (21.3%)	26 (8.2%)	**0.002**
Troponin dynamic changes, n (%)	21 (41.2%)	45 (18.7%)	** < 0.001**
Baseline blood glucose, mmol/L (IQR)	7.26 (6.27–9.58)	7.09 (6.00–9.03)	0.47
Platelet, 10^9^/L (IQR)	190 (157–218)	193 (167–237)	0.096
INR (IQR)	1.06 (1.03–1.12)	1.03 (0.97–1.09)	**0.001**
Creatinine, umol/L (IQR)	70 (61–91)	70 (60–85)	0.49
HbA1c,% (IQR)	5.80 (5.47–6.26)	5.90 (5.49–6.60)	0.52
TC, mmol/L (IQR)	4.01 (3.51–4.59)	4.33 (3.66–5.11)	0.12
LDL_C, mmol/L (IQR)	2.43 (1.87–3.13)	2.60 (2.05–3.32)	0.18
**Stroke etiology**			** < 0.001**
Large artery atherosclerosis	15 (24.6%)	96 (30.4%)	
Cardioembolism	35 (57.4%)	91 (28.8%)	
Small vessel occlusion	2 (3.3%)	76 (24.1%)	
Other determined	3 (4.9%)	7 (2.2%)	
Undetermined	6 (9.8%)	46 (14.6%)	

*DBP, diastolic blood pressure; HT, hemorrhagic transformation; INR, international normalized ratio; IQR, interquartile range; LDL-C, low-density lipoprotein cholesterol; NIHSS, National Institutes of Health Stroke Scale; OTT, onset-to-treatment time; SBP, systolic blood pressure; SD, standard deviation; TC, total cholesterol. The bold values represent significant results in univariate analysis (*P* < 0.05).*

Univariate logistic regression analysis showed that both patients with troponin elevation on admission (OR 3.02, 95%CI 1.45–6.28, *p* = 0.003; Table 3) and troponin dynamic changes (OR 3.05, 95%CI 1.60–5.81, *p* = 0.001; Table 3) had a higher risk of HT. This association stabilized in model 1 which was adjusted for age and sex. Whereas in model 2, troponin dynamic changes were independently positively associated with HT risk (OR 2.27, 95%CI 1.06–4.85, *p* = 0.034; Table 3) and troponin elevation on admission had an insignificantly trend to develop HT (OR 2.23, 95%CI 0.96–5.21, *p* = 0.063; Table 3). Subgroup analysis showed that troponin elevation on admission in the elderly was related to HT risk, while troponin dynamic changes were associated with HT risk in the patients with advanced age, AF and higher stroke severity (Supplementary Tables 3, 4). Rising troponin dynamic changes (OR 2.20, 95%CI 1.05–4.60, *p* = 0.037), rather than troponin elevation, were associated with unfavorable outcome at discharge when confounding factors were considered (Supplementary Table 5).

**TABLE 3:** Logistic regression analysis to identify relationships between troponin elevation on admission, troponin dynamic changes and HT, respectively.

	**Crude model**		**Model 1**		**Model 2**	
	**OR (95%CI)**	***P***	**OR (95%CI)**	***P***	**OR (95%CI)**	***P***
Troponin elevation on admission	3.02 (1.45–6.28)	0.003	2.96 (1.42–6.21)	0.004	2.23 (0.96–5.21)	0.063
Troponin dynamic changes	3.05 (1.60–5.81)	0.001	3.00 (1.54–5.85)	0.001	2.27 (1.06–4.85)	0.034

*Model 1: adjusted for age and sex.*

*Model 2: adjusted for age, sex, atrial fibrillation, NIHSS score, ejection fraction, platelet, INR and stroke etiology.*

*CI, confidence interval; HT, hemorrhagic transformation; INR, international normalized ratio; NIHSS, National Institutes of Health Stroke Scale; OR, odds ratio.*

## Discussion

Our study revealed the association of elevated troponin levels, especially its dynamic changes, with HT after thrombolysis in 377 patients with AIS. In contrast to troponin elevation on admission, troponin dynamic changes were more common in patients receiving thrombolysis and were dominated by an ascending pattern. And troponin dynamic changes were also more strongly associated with the risk of HT after thrombolysis than the former, corresponding results could still be observed in part of the subgroups.

Troponin elevation is common in patients with AIS, and causes of troponin elevation can be divided into acute myocardial injury and chronic myocardial injury based on whether dynamic changes are observed with serial troponin assays (Scheitz et al., 2021). Unlike previous studies (Anders et al., 2013; Scheitz et al., 2014), troponin dynamic changes accounted for a greater proportion of patients with elevated troponin levels in our study. This may be result from the fact that the time point of our first measurement was within 4.5 h, and the median time interval for follow-up measurement was 24 h. Besides, differences in the definition of troponin dynamic changes, troponin assay for detection and race may contribute to the discrepancies.

Chronic myocardial injury is mostly caused by pre-existing heart diseases; renal insufficiency also reflects coexisting serious heart diseases (Scheitz et al., 2021). In addition to the comorbid ACS, acute myocardial injury is usually owing to stroke–heart syndrome (Scheitz et al., 2018, 2021). Stroke-heart syndrome is in virtue of acute cerebral ischemia injury to the central autonomic network, which then leads to activation of the sympathetic nervous system and the hypothalamic–pituitary–adrenal axis. The activation will bring excessive release of catecholamine and cortisol, ultimately predisposing ischemic and non-ischemic myocardial injury. Stroke-heart syndrome is not only related to stroke severity and location, but also occur more frequently in patients in whom chronic myocardial injury is present, thereby further amplifying acute injury. The presence of troponin elevation and dynamic changes observed in our study was associated with advanced age, comorbid heart disease, renal dysfunction, and more severe clinical symptoms, which is in line with the above theory. Therefore, serial troponin assays should be intensified in thrombolytic patients with the above risk factors. In addition, enhanced assay is equally required in patients with troponin elevation on admission.

Hemorrhagic transformation (HT) is a common adverse reaction of thrombolytic therapy, and the main mechanism involves blood-brain barrier disruption (Arba et al., 2020). In a small sample study, troponin elevation measured within 48 h in patients with AIS attributed to rheumatic heart disease was found to be associated with HT (Liu et al., 2016a). This is a cross-sectional study that cannot establish a causal association of troponin elevation with HT. And the study targets a special group of patients with rheumatic heart disease, which is not a common cause of stroke. By comparison, thrombolysis is widely used and HT is desirable to be avoided. Our study found that troponin elevation on admission was associated with HT after thrombolytic therapy, whereas it showed a non-significant tendency after adjusting for confounders. On the one hand, it may be explained by the fact that troponin elevation is often associated with advanced age, AF, and higher NIHSS score, which are all risk factors for HT (Álvarez-Sabín et al., 2013). On the other hand, since subclinical myocardial damage and brain damage tend to coexist (Russo et al., 2013a,b), troponin, used as an indicator of response to myocardial injury, may also represent underlying brain injury. Up to now, troponin elevation has been found to be related to cerebral small vessel disease including white matter hyperintensities, cerebral microbleeds (Dadu et al., 2013; Liu et al., 2016b; von Rennenberg et al., 2019). Moreover, cerebral small vessel disease primarily induced by blood-brain barrier disruption (Li et al., 2018) and is closely associated with HT after thrombolysis (Willer et al., 2015; Nagaraja et al., 2018). Therefore, blood-brain barrier disruption may be a potential mechanism mediating troponin elevation and HT after thrombolysis.

Compared with troponin elevation on admission, the association between troponin dynamic changes and HT after thrombolysis was stronger. Rising troponin dynamic changes may also reflect stroke-heart syndrome caused by HT. It is reported that nearly 90% of HT after thrombolysis occurs within 24 h after treatment (Strbian et al., 2011), while the median time of follow-up troponin assay in our study was beyond 24 h. However, this is not applicable to the falling pattern. None of patients with falling troponin dynamic changes presented with PH. Falling troponin dynamic changes, similar to troponin elevation on admission, should be related to HI through the blood-brain barrier breakdown and the concomitant clinical risk factors of HT. Therefore, in patients with troponin elevation on admission, the possibility of HT cannot be excluded even if a decline in troponin levels was observed on serial measurements.

We found prognostic significance of troponin for unfavorable outcome at discharge in patients with AIS. A recent meta-analysis provided compelling evidence that troponin elevation can predict all-cause mortality in AIS (Fan et al., 2018). Previous studies have shown that patients with troponin elevation have poor functional outcome at discharge (Scheitz et al., 2012, 2014), and troponin dynamic changes are related to in-hospital mortality (Scheitz et al., 2014). In our study, rising dynamic changes observed by serial measurements were more prone to poor outcome at discharge than troponin elevation shown by a single assay on admission. This is perhaps because the rising changes are in response to worsening cerebral lesions.

The study still has limitations. Firstly, previous study has found that approximate 25% AIS patients with troponin elevation of at least 4-fold URL have acute coronary culprit lesions (Mochmann et al., 2016). As for our study, ACS in patients with elevated troponin levels was excluded mainly through symptoms and electrocardiogram changes rather than coronary angiography. But because quite a few patients have aphasia or impaired consciousness, ACS may be underestimated. Secondly, although we performed serial measurement in troponin, nearly a quarter of the patients were detected only once. It is also questionable whether the measurement interval is appropriate and the number of times is adequate. Thirdly, we converted troponin into a categorical variable for analysis due to the existence of the lower limit of detection, which may have attenuated the association with HT. Fourthly, whether blood-brain barrier disruption is the mediator of the troponin elevation on admission and HT was not directly validated, but inferred from the results of previous studies. However, after classifying the troponin dynamic changes into rising and falling types, we found that the relationship between rising dynamic changes and HT is different from falling dynamic changes and troponin elevation on admission. Finally, as a single center, moderate sample study, our results still need further confirmation, especially that the association between troponin elevation on admission and HT was not significant after adjustment.

## Conclusion

Overall, we found that troponin dynamic changes including both rising and falling pattern were independently associated with HT after thrombolysis. Rising troponin dynamic changes were also prone to adverse outcomes at discharge. Patients with advanced age, premorbid heart disease, renal insufficiency, higher NIHSS score, and troponin elevation on admission tend to present troponin dynamic changes. Continuous monitoring for troponin levels in thrombolytic patients with relevant risk factors is warranted.

## Data Availability Statement

The raw data supporting the conclusions of this article will be made available by the authors, without undue reservation.

## Ethics Statement

The studies involving human participants were reviewed and approved by the Ethics Committee of The Second Affiliated Hospital and Yuying Children’s Hospital of the Wenzhou Medical University. The patients/participants provided their written informed consent to participate in this study.

## Author Contributions

ZH and ZC conceived and designed the study. ZC and ZZ analyzed the data. ZC, ZZ, and XH assisted in data interpretation. ZC wrote the manuscript. All authors acquired the data, participated in revising the article, and approved the final version.

## Conflict of Interest

The authors declare that the research was conducted in the absence of any commercial or financial relationships that could be construed as a potential conflict of interest.

## Publisher’s Note

All claims expressed in this article are solely those of the authors and do not necessarily represent those of their affiliated organizations, or those of the publisher, the editors and the reviewers. Any product that may be evaluated in this article, or claim that may be made by its manufacturer, is not guaranteed or endorsed by the publisher.
