# Supplementary material for: Troponin Elevation on Admission Along With Dynamic Changes and Their Association With Hemorrhagic Transformation After Thrombolysis
Source: Front Aging Neurosci. 2021 Oct 13;13:758678. doi: 10.3389/fnagi.2021.758678 (PMC8548361; doi:10.3389/fnagi.2021.758678)
Supplement: Supplementary file 1 [file Data_Sheet_1.docx]

Supplementary Material

# Supplementary Figures and Tables

Table S1. Clinical characteristics of patients, stratified by the presence of serial measurements in troponin

|  | Serial measurements in troponin | | *P* |
| --- | --- | --- | --- |
|  | Yes (n=291) | No (n=86) |  |
| Age, years (IQR) | 71 (62-81) | 70 (59-78) | 0.22 |
| Male, n (%) | 191 (65.6%) | 59 (68.6%) | 0.61 |
| Vascular risk factors | | | |
| Hypertension, n (%) | 224 (77.0%) | 65 (75.6%) | 0.79 |
| Diabetes mellitus, n (%) | 93 (32.0%) | 22 (25.6%) | 0.26 |
| Hyperlipidemia, n (%) | 108 (37.1%) | 29 (33.7%) | 0.57 |
| Current Smoking, n (%) | 68 (23.4%) | 22 (25.6%) | 0.67 |
| Comorbidities | | | |
| Atrial fibrillation, n (%) | 103 (35.4%) | 24 (27.9%) | 0.20 |
| Coronary artery disease, n (%) | 29 (10.0%) | 1 (1.2%) | **0.008** |
| Previous stroke, n (%) | 36 (12.4%) | 9 (10.5%) | 0.63 |
| Treatment status | | | |
| OTT, min (mean±SD) | 165.3±56.3 | 163.2±59.6 | 0.77 |
| Ongoing antithrombotic therapy, n (%) | 44 (15.1%) | 9 (10.5%) | 0.28 |
| Bridge therapy, n (%) | 42 (14.4%) | 6 (7.0%) | 0.068 |
| NIHSS score (IQR) | 8 (5-15) | 6 (4-12) | **0.039** |
| Baseline SBP, mmHg (mean±SD) | 159.9±23.2 | 162.4±26.7 | 0.39 |
| Baseline DBP, mmHg (mean±SD) | 88.8±16.6 | 91.2±16.9 | 0.34 |
| Ejection fraction,% (IQR) | 64 (60-67) | 64 (62-68) | 0.19 |
| HT, n (%) | 51 (17.5%) | 10 (11.6%) | 0.19 |
| Laboratory test | | | |
| Troponin elevation on admission, n (%) | 33 (11.3%) | 6 (7.0%) | 0.24 |
| Baseline blood glucose, mmol/L (IQR) | 7.09 (6.04-9.22) | 7.19 (6.02-8.98) | 0.94 |
| Platelet, 10^9^ /L (IQR) | 190 (163-227) | 204 (172-248) | **0.017** |
| INR (IQR) | 1.03 (0.98-1.09) | 1.03 (0.97-1.08) | 0.55 |
| Creatinine, umol/L (IQR) | 71 (60-86) | 70 (60-84) | 0.71 |
| HbA1c,% (IQR) | 5.90 (5.50-6.50) | 5.71 (5.44-6.33) | 0.24 |
| TC, mmol/L (IQR) | 4.21 (3.68-5.06) | 4.35 (3.52-5.05) | 0.60 |
| LDL_C, mmol/L (IQR) | 2.54 (2.05-3.31) | 2.64 (1.87-3.17) | 0.64 |

DBP, diastolic blood pressure; HT, hemorrhagic transformation; INR, international normalized ratio; IQR, interquartile range; LDL-C, low-density lipoprotein cholesterol; NIHSS, National Institutes of Health Stroke Scale; OTT, onset-to-treatment time; SBP, systolic blood pressure; SD, standard deviation; TC, total cholesterol.

Table S2. Clinical characteristics of patients, stratified by the presence of troponin elevation on admission

|  | Troponin elevation on admission | | *P* |
| --- | --- | --- | --- |
|  | Yes (n=39) | No(n=338) |  |
| Age, years (IQR) | 78 (65-83) | 70 (61-80) | 0.063 |
| Male, n (%) | 21 (53.8%) | 229 (67.8%) | 0.082 |
| Vascular risk factors | | | |
| Hypertension, n (%) | 34 (87.2%) | 255 (75.4%) | 0.101 |
| Diabetes mellitus, n (%) | 8 (20.5%) | 107 (31.7%) | 0.15 |
| Hyperlipidemia, n (%) | 15 (38.5%) | 122 (36.1%) | 0.77 |
| Current Smoking, n (%) | 8 (20.5%) | 82 (24.3%) | 0.60 |
| Comorbidities | | | |
| Atrial fibrillation, n (%) | 22 (56.4%) | 105 (31.1%) | **0.002** |
| Coronary artery disease, n (%) | 8 (20.5%) | 22 (6.5%) | **0.006** |
| Previous stroke, n (%) | 7 (17.9%) | 18 (11.2%) | 0.34 |
| Treatment status | | | |
| OTT, min (mean±SD) | 156.7±57.4 | 165.8±57.0 | 0.35 |
| Ongoing antithrombotic therapy, n (%) | 9 (23.1%) | 44 (13.0%) | 0.087 |
| Bridge therapy, n (%) | 2 (5.1%) | 46 (13.6%) | 0.13 |
| NIHSS score (IQR) | 14 (9-18) | 7 (4-13) | **<0.001** |
| Baseline SBP, mmHg (mean±SD) | 160.3±23.2 | 160.5±24.1 | 0.97 |
| Baseline DBP, mmHg (mean±SD) | 93.4±20.5 | 88.9±16.1 | 0.11 |
| Ejection fraction,% (IQR) | 51 (58-66) | 64 (61-68) | **0.001** |
| HT, n (%) | 13 (33.3%) | 48 (14.2%) | **0.002** |
| Laboratory test | | | |
| Troponin dynamic changes, n (%)^†^ | 26 (78.8%) | 40 (15.4%) | **<0.001** |
| Baseline blood glucose, mmol/L (IQR) | 6.95 (5.90-8.31) | 7.15 (6.06-9.28) | 0.32 |
| Platelet, 10^9^ /L (IQR) | 198 (159-232) | 191 (165-233) | 0.65 |
| INR (IQR) | 1.06 (1.00-1.12) | 1.03 (0.98-1.09) | 0.079 |
| Creatinine, umol/L (IQR) | 84 (69-110) | 69 (59-84) | **<0.001** |
| HbA1c,% (IQR) | 5.90 (5.50-6.34) | 5.85 (5.49-6.60) | 0.92 |
| TC, mmol/L (IQR) | 4.23 (3.53-5.20) | 4.24 (3.66-5.05) | 0.84 |
| LDL_C, mmol/L (IQR) | 2.39 (2.01-3.45) | 2.57 (2.03-3.28) | 0.93 |
| Stroke etiology |  |  | **0.008** |
| Large artery atherosclerosis | 9 (23.1%) | 102 (30.2%) |  |
| Cardioembolism | 23 (59.0%) | 103 (30.5%) |  |
| Small vessel occlusion | 5 (12.8%) | 73 (21.6%) |  |
| Other determined | 0 (0.0%) | 10 (3%) |  |
| Undetermined | 2 (5.1%) | 50 (14.8%) |  |

DBP, diastolic blood pressure; HT, hemorrhagic transformation; INR, international normalized ratio; IQR, interquartile range; LDL-C, low-density lipoprotein cholesterol; NIHSS, National Institutes of Health Stroke Scale; OTT, onset-to-treatment time; SBP, systolic blood pressure; SD, standard deviation; TC, total cholesterol.

† Data were available in 291 patients.

Table S3. Subgroup analysis to identify relationships between troponin elevation on admission and HT

|  | OR (95% CI) | *P* | *P* for interaction |
| --- | --- | --- | --- |
| Age, years |  |  | 0.27 |
| ≤ 70 | 0.47 (0.065-3.46) | 0.46 |  |
| > 70 | 3.67 (1.28-10.49) | 0.015 |  |
| Sex |  |  | 0.063 |
| Male | 1.25 (0.38-4.07) | 0.72 |  |
| Female | 3.21 (0.73-14.09) | 0.12 |  |
| Atrial fibrillation |  |  | 0.73 |
| No | 1.31 (0.23-7.43) | 0.76 |  |
| Yes | 2.88 (0.99-8.40) | 0.053 |  |
| NIHSS score |  |  | 0.35 |
| ≤ 8 | 2.12 (0.25-18.29) | 0.49 |  |
| > 8 | 1.69 (0.65-4.39) | 0.28 |  |

Adjusted for the variables in model 2 except for the grouping variables.

CI, confidence interval; HT, hemorrhagic transformation; NIHSS, National Institutes of Health Stroke Scale; OR, odds ratio.

Table S4. Subgroup analysis to identify relationships between troponin dynamic changes and HT

|  | OR (95% CI) | *P* | *P* for interaction |
| --- | --- | --- | --- |
| Age, years |  |  | 0.18 |
| ≤ 70 | 1.26 (0.28-5.60) | 0.76 |  |
| > 70 | 3.24 (1.22-8.62) | 0.018 |  |
| Sex |  |  | 0.47 |
| Male | 2.16 (0.78-5.97) | 0.14 |  |
| Female | 3.00 (0.67-13.47) | 0.15 |  |
| Atrial fibrillation |  |  | 0.35 |
| No | 1.17 (0.31-4.43) | 0.82 |  |
| Yes | 3.61 (1.28-10.22) | 0.016 |  |
| NIHSS score |  |  | 0.95 |
| ≤ 8 | 0.27 (0.014-5.20) | 0.39 |  |
| > 8 | 2.65 (1.11-6.30) | 0.028 |  |

Adjusted for the variables in model 2 except for the grouping variables.

CI, confidence interval; HT, hemorrhagic transformation; NIHSS, National Institutes of Health Stroke Scale; OR, odds ratio.

Table S5. Logistic regression analysis to identify relationships between troponin elevation on admission, troponin dynamic changes and unfavorable outcome, respectively

|  | Crude Model |  | Adjusted Model |  |
| --- | --- | --- | --- | --- |
|  | OR (95%CI) | *P* | OR (95%CI) | *P* |
| Troponin elevation on admission | 2.92 (1.43-5.97) | 0.003 | 1.83 (0.79-4.24) | 0.16 |
| Troponin dynamic changes | 3.29 (1.82-5.96) | <0.001 | 1.73 (0.85-3.52) | 0.13 |
| Rising troponin dynamic changes | 3.86 (2.05-7.27) | <0.001 | 2.20 (1.05-4.60) | 0.037 |

Adjusted for age, sex, HT, atrial fibrillation, bridge therapy, NIHSS score, baseline SBP, baseline DBP, baseline blood glucose, ejection fraction, and stroke etiology.

CI, confidence interval; DBP, diastolic blood pressure; HT, hemorrhagic transformation; NIHSS, National Institutes of Health Stroke Scale; OR, odds ratio; SBP, systolic blood pressure.


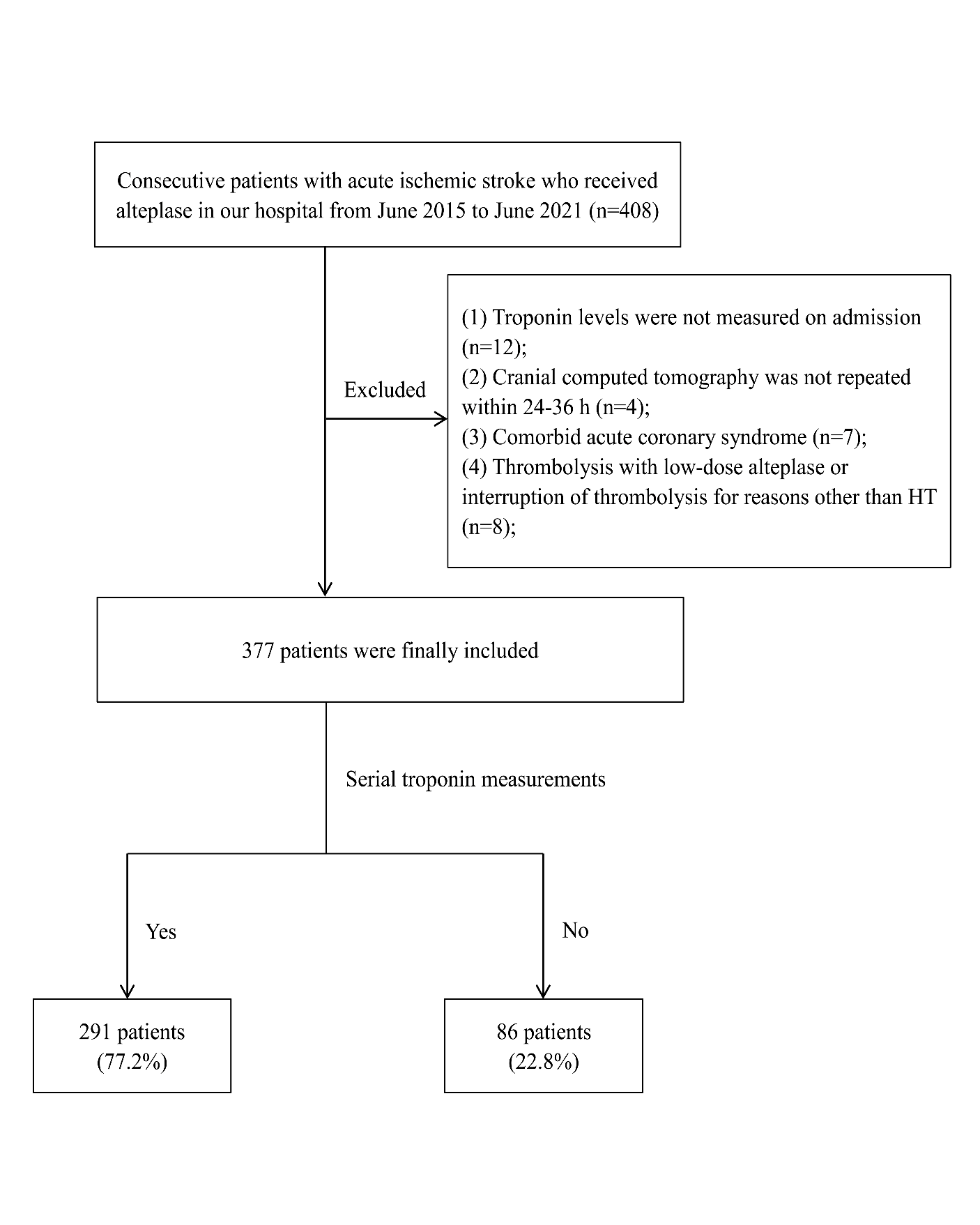
Figure S1. Protocol flow chart in this study.
